# Assessment of Influential Factors for Scours Associated with *Cryptosporidium* sp., Rotavirus and Coronavirus in Calves from Argentinean Dairy Farms

**DOI:** 10.3390/ani11092652

**Published:** 2021-09-09

**Authors:** Emiliano Bertoni, Adrián A. Barragán, Marina Bok, Celina Vega, Marcela Martínez, José F. Gil, Rubén O. Cimino, Viviana Parreño

**Affiliations:** 1 Área de Investigación en Salud Animal, IIACS-CIAP, INTA EEA Salta, Cerrillos A4403, Argentina; bertoni.emiliano@inta.gob.ar; 2 Veterinary Extension, Field Investigation & Research, Department of Veterinary and Biomedical Sciences, The Pennsylvania State University, State College, PA 16801, USA; axb779@psu.edu; 3 Instituto de Virología e INCUINTA, CICV y A, INTA Buenos Aires, Castelar 1712, Argentina; bok.marina@inta.gob.ar (M.B.); vega.celina@inta.gob.ar (C.V.); 4 Área de Producción Animal, INTA EEA Salta, Cerrillos A4403, Argentina; martinez.gabriela@inta.gob.ar; 5 Cátedra de Química Biológica, Facultad de Ciencias Naturales, Universidad Nacional de Salta, Salta A4400, Argentina; jgil.unsa@gmail.com (J.F.G.); rubencimino@gmail.com (R.O.C.); 6 Instituto Nacional de Tecnología Agropecuaria, CICVyA, INCUINTA, Nicolas Repetto y de los Reseros s/n, Buenos Aires 1686, Argentina

**Keywords:** influential factors in dairy calves, scours, *Cryptosporidium* sp., rotavirus, coronavirus

## Abstract

**Simple Summary:**

Scours is the most common disease in dairy calves, and it is a multifactorial syndrome complex. *Cryptosporidium* sp., rotavirus group A, and bovine coronavirus are the three main pathogens associated with scours. The objective of this study was to identify potential factors associated with scours and these three pathogens in preweaned dairy calves. The results of this study indicated that scours is a prevalent disease in farms of Salta, Argentina, and that rotavirus and *Cryptosporidium* sp. infections, along with specific farm management practices, might be important contributing factors that could increase the chance of scours in dairy farms.

**Abstract:**

Scours is the most common disease in dairy calves, and it is a multifactorial syndrome complex. *Cryptosporidium* sp. (C. sp.), rotavirus group A (RVA), and bovine coronavirus (BCoV) are the three main pathogens associated with scours. The objective of this study was to identify potential factors associated with scours, C. sp., RVA, and BCoV infections in preweaned dairy calves from Lerma Valley in Salta Province, Argentina. A total of 488 preweaned calves from 19 dairy farms located in the Lerma Valley were enrolled in this observational study. One fecal sample was collected from each calf between one week and two months of age for assessment of C. sp., RVA, and BCoV infection status. *Cryptosporidium* sp. oocysts and RVA and BCoV antigens in fecal samples were assessed using microscopic observation and indirect enzyme-linked immune sorbent assay (iELISA), respectively. A voluntary questionnaire was developed and used to collect data regarding management practices from the participants’ farms. The data were analyzed using multivariable logistic regression models. Scours incidence was 35.4%, and a greater proportion of calves younger than 20 days were affected. Of the fecal samples, 18% and 9.5% tested were positives for C. sp. and RVA, respectively, while BCoV was detected only in two calves. Furthermore, 84.2% and 63.1% of the farms tested positive for *Cryptosporidium* sp. and RVA, respectively. In addition, the following variables were associated with higher odds of having scours: (1) herd size (>300 milking cows; OR = 1.7), (2) calf age (<20 days of age; OR = 2.2), (3) RVA and C. sp. test (positive test; RVA OR = 2.6; C. sp. OR = 3), calf feeding practices (feeding milk replacer; OR = 1.81), and newborn calf management practices (calf moved from maternity pen <6 h after calving; OR = 1.7). Concerning RVA infection, calves less than 20 days of age (OR = 2.6) had a higher chance of testing positive for RVA, while calves that remained in the calving pen for less than 6 h after calving had a lower chance (OR = 0.3). On the other hand, for C. sp. infection, large farm size (>300 milking cows; OR = 1.2) and young calf age (<20 days of age; OR = 4.4) indicated a higher chance of testing positive for C. sp., while calves belonging to farms that fed frozen colostrum (OR = 0.2) had a lower chance of becoming infected with C. sp. The result of this study indicated that scours is a prevalent disease in farms of the Lerma Valley, Salta, Argentina, and that RVA and C. sp. infections, along with specific farm management practices, might be important contributing factors that could increase the chance of NCS in dairy farms.

## 1. Introduction

Scours is defined as the presence of semiliquid or liquid stool in preweaned calves (Physical appearance code 3 and 4 [1]) and is one of the most common diseases in dairy calves, causing important economic losses for dairy production worldwide [2]. This disease is a multifactorial syndrome affected by several factors including the host (immunological and nutritional status), the pathogens (virus, protozoa, and bacteria), and the environment (climate, housing, hygienic conditions) [3].

*Cryptosporidium* sp. (C. sp.), Rotavirus serotype A (RVA), and Bovine Coronavirus (BCoV) are the three main pathogens associated with scours [4,5], and they are widely distributed throughout the world [6,7]. Several surveys reported these pathogens as causal agents of scours in calves in mono infections and mixed infections [8,9,10,11,12,13,14,15].

A better understanding of the relationship between the presence of pathogens, the management practices, and the health of newborn calves will help to improve the husbandry conditions, and subsequently, avoid or reduce morbidity, mortality, and economic losses associated with scours in dairy farms [16,17]. The multifactorial nature of scours makes the identification of individual disease-contributing factors challenging. Thus, it is crucial to assess, and address, all possible contributing factors when implementing control strategies to prevent or manage scours in dairy farms [11,18]. 

Although scours is a prevalent disease in Argentina, there is a lack of published epidemiological studies aimed at identifying contributing factors for scours in dairy farms. Therefore, the objective of this study was to identify potential factors associated with scours, C. sp., RVA, and BCoV infections in preweaned dairy calves from the northwest region of Argentina.

## 2. Materials and Methods

### 2.1. Geographic Area and Productive Characteristics 

The Lerma Valley is located in the Salta province, Northern Argentina, between 24°30′ S and 25°37′ S and between 65°22′ W y 65°40′ W, with an extension of 17,000 km^2^ to a height of 1100 to 1450 m over the sea level. It has temperate, humid weather (subtropical climate); the valley descends from west to east with a marked dry season that lasts from April to the beginning of October. The rainy season is in spring and summer (between November and December), and the dry season is in autumn and winter (between May and October [19]). The average annual rainfall is 900 mm per year, with a maximum in the summer or wet season. The average temperature is about 23 °C (74 °F) in summer and 15 °C (60 °F) in winter, the dry season. The calving season starts in the fall and ends towards the end of the winter, so the climatic conditions of these two seasons are similar. In the Lerma Valley, there are a total of 51 dairy herds housing 6575 dairy cows, producing around 102,564 kilos of milk per day [20]. The average herd size for this region is around 200 milking cows [20]. Most of these operations are grazing systems, of which the primary diet is pasture-based with grain and mineral mix supplementation [19].

### 2.2. Animal Enrollment and Facilities

This cross-sectional study was performed between 2014 and 2016 during the calving seasons (between April and November). A total of 488 individual fecal samples from calves younger than two months of age with and without scours (defined as the presence of semi-liquid or liquid stool in pre-weaned calves; Physical appearance code 3 and 4 [1]) were collected using the convenience sample collection method [21] from 19 dairy farms. The study team visited each farm one or two times during the study period to collect information regarding possible factors associated with scours. Furthermore, the scours dam vaccination protocol consisted of vaccines against rotavirus and *Escherichia coli* administered around 60 d and 30 d before the expected calving date, and only two farms measured colostrum quality as part of their colostrum administration protocols. 

The herd’s size was categorized as small (<200; *n* = 6), medium (200–300; *n* = 8), or large (>300 milking cows; *n* = 5) depending on the amount of milking cows. Concerning the prepartum management, around 21 days before the expected calving date, pregnant cows were moved into a group dry lot calving pen (*n* = 10). Cows were monitored for signs of parturition, such as the appearance of the water bag or calf’s feet through the vulva, every 6 h. Cows showing signs of difficult calvings were assisted following the Standard Operating Procedures (SOPs). Calves remained with the dam in the group pens for less than 6 h from calving (*n* = 5) or more than 6 h (*n* = 14), where they were bottle or tube-fed colostrum (*n* = 6) or were allowed to nurse from the dam (*n* = 13). After calves received the first colostrum, they were moved from the calving pen to individual stakes (*n* = 11) or group pens (~10 calves per pen; *n* = 8). Feeding until weaning consists of two liters of raw milk (*n* = 8) or milk substitute (*n* = 11) in the morning and the afternoon fed in buckets, plus supplement prestarter ad libitum starting in the first week of life. Health monitoring was carried out during the feeding routine by the caretaker twice a day. Weaning was performed based on two months of life. 

### 2.3. Assessment of Farm Management Practices 

A standard voluntary multiple-choice survey was developed to collect information about preweaned calf management practices in the participant farms. Questions about the following were included in this survey: (1) herd size (small <200, medium 200–300, or large >300 milking cows), (2) the timing for moving newborn calves out of maternity pens (<6 h, 7–11 h, 24–47 h, or 48–72 h), (3) navel disinfection (yes or no), (4) colostrum intake (nursing, bottle or esophageal tube), (5) the volume of colostrum intake (unknown, <4 L, or >4 L), (6) feeding frozen colostrum (yes or no), (7) calf rearing system (individual or group), (8) pre-weaned calf feeding (milk replacer or raw milk), (9) number of caretakers per 25 calves (one, two, or three), (10) caretakers’ gender (female or male), (11) work shift length (4 h or 8 h), and (12) scours dam vaccination (yes or no). 

The surveys were completed by the farm veterinarian. Briefly, veterinarians from the Lerma Valley were contacted through phone calls or email. Veterinarians’ contact information was obtained through the Heath Animal Area of the Instituto Nacional de Tecnología Agropecuaria (INTA) database. Out of the 7 veterinarians that were contacted, 5 practitioners, who provided animal health services to 19 dairy farms, agreed to participate in this study. 

### 2.4. Samples Collection and Analysis 

Approximately 10 g of feces were collected directly from the rectum of calves using a sterile plastic bag, placed on a cooler with ice, and transported to the laboratory, where samples were kept at 4 °C until processed within 24 h from collection. Aliquots of each fecal sample were diluted 10% (*w*/*v*) in phosphate saline buffer solution (pH 7) and conserved at −20 °C or −70 °C until bovine RVA and bovine BCoV analyses, respectively. Another aliquot was diluted 5% (*w*/*v*) in formaldehyde solution for the detection of C. sp. oocysts. 

Polyclonal indirect enzyme-linked immune sorbent assay (iELISA) was performed for RVA antigen detection as previously described elsewhere [22,23]. A monoclonal iELISA was used for BCoVB antigen detection with a sensitivity of 97.2% and a specificity of 100% [24]. For the observation of C. sp. oocysts, 7.5 mL of 5% diluted aliquoted fecal samples was centrifugated at a 7200× *g* for five minutes, the supernatant was discharged, and an aliquot was taken from the sediment surface with a laboratory handle to perform fecal smears that were dyed with the Ziehl–Neelsen modified technique. Those samples where more than two structures were compatible with oocytes of C. sp. (circular structures stained red with a size about 4–6 um) were considered positive [25]. 

### 2.5. Statistical Analysis

Using the ProMESA 1.2 software with a 95% confidence interval, standard error of 20%, and considering a scours incidence of 15% (estimated based on informal communication with 10 local veterinarians), it was estimated that a minimum of 474 animals would be required to successfully complete this experiment. 

The factor (odds ratio) analysis was conducted using the Epical package (http://cran.r-project.org (accessed on: 8 April 2019)) in R Studio (Version 1.2.1335, © 2009–2019 RStudio, Inc., Boston, MA, USA). A first univariate screening of variables was conducted in order to identify significant factors associated with diarrhea, rotavirus infection, and C. sp. infection. The Epicalc function “*cc*” was applied, producing odds ratio, its 95% confidence interval, and performing the Chi-squared (χ2) tests and Fisher’s exact tests. The ‘*cc*’ function uses the exact method to calculate the odds ratio (Epical Manual, https://usermanual.wiki/Document/EpicalcBook.934243917/view (accessed on: 8 April 2019)). Due to the low incidence of BCoV in the study population (2 calves positive), this variable was excluded from the analysis.

The variables with a *p* < 0.2 [11,26] in the univariate analysis were selected as explanatory effects for performing a Multivariable Logistic Regression (MLR) model where calf within farm was included in the model as a random effect. Using the Epicalc command “*logistic. display*”, a display showing the OR (95% CI), *p* (Wald’s test), and *p* (LR-test) was obtained. For the interpretation of the results, it was considered that the *p*-value obtained in the Wald’s test depended on the reference level of the explanatory variable with only two categories, while the *p*-value from LR-test was considered in the analysis of the variables with tree or more categories. 

The model selection was conducted by stepwise selection of independent variables, we started with a full model including all variables selected with the univariate screening (*p* < 0.2). The command step removed each independent variable and compared the degrees of freedom reduced, the new deviance, and the new AIC. The results were increasingly sorted by AIC. R selected the best fit model with the lowest Akaike Information Criterion (AIC) score [11,27]. The variables of interest were considered significant if *p* < 0.05, and *p* < 0.10 was considered a tendency. 

## 3. Results

### 3.1. Study Population Demographics

A total of 19 dairy farms participated in the present study, representing 37% of the total number of farms in the Lerma Valley. The description of the breed of cows, herd size, calf rearing system, and pre-weaned calf feeding management in the study farms is provided in Table 1. The average calf age for the calves enrolled in the study was 26.8 ± 18.6 (SD) days (Table 1). 

### 3.2. Overall Scours, RVA, BCoV and C. sp. Incidences

**Figure 1:**
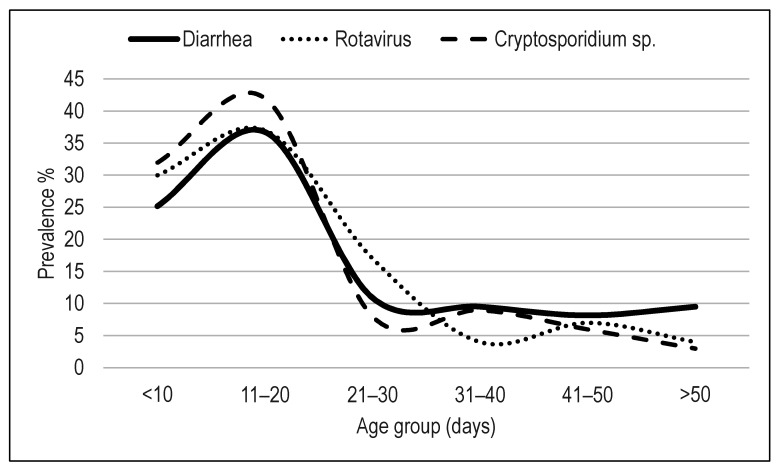
Incidence of scours, rotavirus, and *Cryptosporidium* sp. infections in the different age groups of dairy calves.

In Table 1 and Figure 1, the results of this section are detailed and represented respectively. From the 488 calves sampled in this study, 173 calves had scours at sampling time (incidence of 35.4%), while 315 presented normal stools. *Cryptosporidium* sp. was found in 18.0% of the samples, representing 84.2% of the farms; while 9.5% of fecal samples tested positive for RVA, representing 63.1% of the study farms. In contrast, only two calves from two different farms were shedding BCov.

As can be observed in the Figure 1, both RVA and C. sp. infections were associated with diarrhea in calves younger than 20 days of age.

### 3.3. Variables Associated with Scours

Screening variables to perform the MLR respect scours, RVA and C. sp. infection are presented in the supplementary files in the Tables S1–S3 respectively.

The output of the final model for scours is presented in Table 2. Calves from dairy farms with more than 300 milking cows had 1.7 higher odds (*p* = 0.01) to have scours compared to calves from smaller farms. Also, calves younger than 20 days of age had higher odds (OR = 2.2; *p* < 0.001) of having scours compared to older calves. Having a positive test for RVA (OR = 2.6; *p* = 0.005) or C. sp. (OR = 3; *p* < 0.001) and feeding milk replacer (OR = 1.8; *p* = 0.01) also increased the odds of having scours in the studied calves. Interestingly, calves that were moved out of the maternity pen within 6 h from calving had higher odds (OR = 1.7; *p* = 0.04) of suffering scours than calves that were moved after 6 h from the calving pen.

### 3.4. Variables Associated with RVA Infection

The output of the final model for RVA infection is presented in Table 3. Concerning RVA infection, calves with a female caretaker (OR= 2.4; *p* = 0.004) and less than 20 days of life (OR = 2.6; *p* = 0.004) had higher odds to become infected with RVA. On the other hand, calves reared individually had less chance of being infected (OR= 0.4; *p* = 0.02) than calves reared in collective paddocks.

### 3.5. Variables Associated with C. sp. Infection

The output of the final model for C. sp. infection is presented in Table 4. For C. sp. infection, calves belonging to dairy farms with more than 300 milking cows (OR = 1.2; *p* = 0.01) and calves less than 20 days of age (OR = 4.4; *p* < 0.001) had higher odds of becoming infected with C. sp., while calves belonging to farms that administered frozen colostrum had higher odds of becoming infected with C. sp. (OR = 5; *p* < 0.001).

## 4. Discussion

This study presents the first cross-sectional of influential factors associated to calf scours in dairy farms located in the Salta province in Argentina. This study also studied the influential factors associated with RVA and C. sp. infections. It is relevant to mention that this was not an experimental controlled study, and the results presented in this manuscript should be considered as an observational survey under field conditions to study the situation of a specific region, and associations and interpretation must be done with cautiousness. 

The rate of scours reported in this study was similar to those registered in dairy farms from other regions of Argentina and worldwide [3,11,28,29,30,31,32,33,34]. Bovine RVA and C. sp. were widely spread among the dairy farms studied, and their incidence was similar to other studies performed in Argentina [13,35,36,37] and worldwide [3,9,11,13,35,36,38,39,40]. The incidence of RVA in dairy calves with and without scours was similar to those reported in studies performed in Brazil and Algeria [41,42], but lower than other studies performed in Iran, Holland, Australia, New Zealand [3,11,39,43], and other Argentinean regions [35,43]. Regarding the worldwide incidence of C. sp. in calves, a wide range from 0.9% to 93% incidence has been described [44,45,46,47,48,49,50]. In respect of the central region of Argentina, the incidence of C. sp. in our study was similar to the results described by Del Coco et al. (2008; 17% [51]) and Tiranti et al. (2011; 19.3% [36]), but lower to the study conducted by Garro et al. (2021; 29% [13]). The prevalence of mix-infection of RVA-C. sp. in calves with or without scours was lower (2.8%) with respect to that reported by Brunauer et al. (6.69% [14]), and Conrady et al. (9.3% [15]), but this comparison must be taken with cautiousness given that the latter’s are worldwide prevalences from a meta-analysis including dairy and beef farms, where studies form Argentina were not included. The variance of the prevalence of the mono and mixed infections could be explained from a geographical influence, like it had been described in the two mentioned meta-analyses [14,15], where the environment and the management system adopted by the farms could act as positive or negative risk factors.

In reference to our low prevalence of coronavirus detection, one of the possible reasons could be the use of ELISA assays that have a lower sensitivity than molecular techniques, or just be a reality of the region. Previous studies conducted by ELISA in Argentina detected BCoV in 1.71% (92/5365) of the samples corresponding to 5.95% (63/1058) of the diarrhea cases registered in 239 beef and 324 dairy farms. The detection rate of BCoV was significantly higher in dairy than in beef herds: 12.13% (29/239) vs. 4.32% (14/324), respectively [12]. In Uruguay, the overall detection rate of BCoV reported by RT-PCR was 7.8% (64/824): 7.7% (60/782) in dairy cattle and 9.5% (4/42) in beef cattle [52], suggesting that the circulation of BCoV in dairy basins located in the Pampean region of two South American countries like Argentina and Uruguay are similar to that reported in the mentioned meta-analysis, regardless of the diagnosis technique used. The prevalence of this disease in Salta, with subtropical weather, was lower than in the temperate weather like Brazil and Algeria, as mentioned above. 

In this study, calves from farms with more than 300 milking cows were almost 2 times more likely to suffer scours and test positive for C. sp. compared with calves from smaller farms. Similar results were reported in other studies [32,53,54,55]. A possible explanation for this association may be related to overcrowding and lack of frequent individual animal health monitoring practices, often observed in large-size operations [30].

In the current study, calves from farms that did not feed frozen colostrum had de-creased odds of testing positive for C. sp. Colostrum samples of this study were not available to assess its microbiology or the antibody concentration for C. sp. and RVA. Regardless, other surveys reported that maternal antibodies in the colostrum did not protect calves against C. sp. infection [56], while other studies did not find an association regarding this practice and C. sp. infection [8,53], and others did find a protective association [57,58]. A possible explanation could be related to the fact that the farms that did not freeze colostrum were median and small farms, where the overcrowding could be lower (date not showed). It is important to highlight that in contrast to RVA and BCoV, there is no vaccine commercially available for C. sp. that can be administrated to cows during the dry period.

Scours incidence in calves that were fed with milk replacers was higher than in calves fed with raw milk. Forty eight percent of the study farms fed calves only with milk replacer twice a day from 24–48 h after birth until weaning (restricting feeding system, 10% of body weight). In the previous survey by Godden et al. (2005 [59]), they observed that calves fed with pasteurized nonsalable milk had lower odds of having scours than calves being fed with milk replacer. The authors argued that differences in scours incidence were related to the better nutritional quality of the pasteurized nonsalable milk, which could have improved the development of the immune system and the presence of antibodies and nonspecific immunity factors, and given calves a better chance to respond against infectious diseases, compared to calves fed milk replacer. Another important factor related to the restricting feeding system that has been reported is that it only covers maintenance requirements with minimal weight gain [60]. The authors also reported that calves fed with this system had more days with scours in the first weeks of life compared to calves fed with a better amount of food [60]. Based on the latter, in the current study, calves fed with milk replacer may had received poor nutrition, which in turn could have increased the risk for scours in this group of animals. In addition, it has been reported that feeding milk replacer to calves could lead to scours due to other factors, such as deficiency of milk clotting in the abomasum, presence of allergens and antitrypsin factors, and altered proteins, due to the high temperatures of product preparation, proportion of fat, and improper formulation based on calves’ age [61].

In the present study, calves that were moved from the calving pen within 6 h after calving had a higher chance of suffering scours than calves that were moved later. There is contrasting information regarding the benefit of the early separation of the calf from the mother, as it was studied in the reviewed paper of Beaver et al. [61], and they concluded that there is a trend toward of the benefit of the cow–calf rearing with respect to scours and C. sp. infection. On the other hand, in this study we observed that calves rearing individually had a lower chance of being infected with RVA. In a previous study, we observed that calves remaining less than 6 h in the calving pen and rearing in the group pen showed a higher chance of suffering scours. We also observed that even with very high passive maternal antibodies, calves still would get infected with bovine RVA and BCoV in the first two weeks of life. In contrast, calves that spent more time with their dams; even with lower levels of antibodies, when rearing individually, they had a lower chance of diarrhea [60]. These results led us to conclude that the calf rearing system is the most important factor related to scours occurrence, bovine RVA, and BCoV infection, and more important than the time in the calving pen. Therefore, further controlled research studies are needed to identify if this practice is beneficial or not, or in which way it can be influenced by other factors like pre-calving dam vaccination, dam age, dam nutrition, calving monitoring, hygiene in the collection and storage of colostrum, and calf rearing system, among others. In general, individual housing systems are recommended to reduce pathogen exposure and to prevent diseased calves from infecting the rest [62], which coincides with our observations. 

Regarding the caretaker gender, calves that were cared for by females had a higher chance of being infected with RVA. No related studies could be found regarding calf caretaker gender and RVA infection. In the studies by Lundborg et al. [63] and Wudu et al. [30], they did not find relationships between the risk of scours and the caretaker gender or number of caretakers. Klein-Jöbstl et al. [32] established the size of the herd as a risk factor, where the prevalence of scours increased as the herds became larger, but the personnel in charge of calve´s care did not increase proportionally, which could fit perfectly to the situation of the dairy farms of the Lerma Valley. 

In agreement with findings reported by others, calves that tested positive for bovine RVA and C. sp. were more likely to suffer scours compared with calves that tested negative, and calves younger than 20 days of life had a higher chance of being infected with RVA and C. sp. [3,4,8,10,11,13]. Also, calves younger than 20 days of age were 3 times more likely to suffer scours compared with older calves, similar to other surveys [3,32,34].

## 5. Conclusions

The results of this study indicate that scours are a problem in the farms of the Lerma Valley, Salta, Argentina, and that RVA and C. sp. are enteric pathogens widely distributed among herds. Furthermore, large-size herds, calves’ age, and a positive diagnosis for RVA and C. sp. may be possible influential factors for scours. Despite that some specific farm practices may be associated with scours, a holistic approach taking into account the multifactorial nature of this condition must be implemented when troubleshooting scours issues in dairy farms. Also, this study provides useful information for advising the farmers of the Lerma Valley regarding improving management to prevent scours. It is important to educate the veterinarians and farmers to vaccinate the herds and increase colostrum antibodies.

## Figures and Tables

**Table 1:** Descriptive result analysis of the variables included as influential factors from the survey performed between 2014 and 2016.

Descriptive Variables	Result
Number of studied farms	19
Total fecal samples	488
Average of fecal samples collected per farm (SD)	25.7 (±7.8)
Diarrhea incidence	35.4%, 173/488
Average age (days of life) of calves with diarrhea (SD)	26.8 (±18.6)
RVA positive farms	63.1%, 12/19
Calf shedding RVA (with and without diarrhea)	9.5%, 46/488
Calf shedding RVA with diarrhea	58.7%, 27/46 0.55%, 27/488
Average age (days of life) of calves shedding RVA (SD)	18.5 (±12.7)
CoV positive farms	10.5%, 2/19
Calf shedding CoV (with and without diarrhea)	0.4%, 2/488
C. sp. positive farms	84.2%, 16/19
Calf shedding C. sp. (with and without diarrhea)	18.2%, 89/488
Calf shedding C. sp. with diarrhea	71.9%, 64/89 13.1%, 65/488
Average days of life of calves shedding C. sp. (SD)	17.7% (±18.7)
RVA and C. sp. co-infection (with and without diarrhea)	2.8%, 14/488
Standard deviation (SD)	
Calf shedding RVA and C. sp. with diarrhea	92.8%, 13/14

**Table 2:** Adjusted OD obtained by multiple logistic regression for the independent variable “scours” Log-likelihood = −277.8; AIC = 571.7.

Variable	Adjusted OR (95% CI)	*p*-Value (Wald Test)	*p*-Value (LR Test)
Farm size Median	0.7 (0.2; 1.0)	-	0.01
Farm size Large	1.7 (0.8; 3.6)	-	
Time in calving pen, <6 h	1.7 (1.3; 1)	0.04	-
Milk replacer	1.8 (1.1; 2.8)	0.01	-
RVA infection	2.6 (1.3; 5.3)	0.005	-
C. sp. infection	3 (1.8; 5)	<0.001	-
Calf age, <20 days	2.2 (1.4; 3.5)	<0.001	-

**Table 3:** Adjusted OD obtained by multiple logistic regression for the independent variable “RVA infection”. Log-likelihood = −141.5; AIC = 291.0.

Variable	Adjusted OR (95% CI)	*p*-Value (Wald Test)	*p*-Value (LR Test)
Calf rearing system, individual	0.4 (0.2;0.9)	0.02	-
Gender, male	2.4 (1.3;4.6)	0.004	-
Calf age, <20 days	2.6 (1.3; 5.0)	0.004	-

**Table 4:** Adjusted OD obtained by multiple logistic regression for the independent variable “*Cryptosporidium* sp. infection”. Log-likelihood = −208.3219. AIC = 426.6.

Variables	Adjusted OR (95% CI)	*p*-Value (Wald Test)	*p*-Value (Test-LR)
Farm size, Median	0.4 (0.2;0.8)	-	0.005
Farm size, Large	1.2 (0.5;2.7)	-	
Colostrum bank, no	0.2 (0.1;0.4)	<0.001	-
Calf age, <20 days	4.4 (2.7; 7.2)	<0.001	-

## Data Availability

Not applicable.

## Supplementary Materials

The following are available online at https://www.mdpi.com/article/10.3390/ani11092652/s1. Table S1. Risk factor associated with neonatal calf diarrhea in dairy farms from the Valle de Lerma, Salta, Argentina. Table S2: Risk factor associated with RVA infection in dairy farms from the Valle de Lerma, Salta, Argentina. Table S3: Risk factor associated with *Cryptosporidium* sp. infection in dairy farms from the Valle de Lerma, Salta, Argentina. Table S4: Statistics outputs of R.
