# Supplementary material for: Assessment of Influential Factors for Scours Associated with Cryptosporidium sp., Rotavirus and Coronavirus in Calves from Argentinean Dairy Farms
Source: Animals (Basel). 2021 Sep 9;11(9):2652. doi: 10.3390/ani11092652 (PMC8466251; doi:10.3390/ani11092652)
Supplement: Supplementary file 1 [file animals-11-02652-s001.zip › animals-1244470-supplementary-part 1-S1, S2, S3.pdf]

animals

Supplementary Materials

Assessment of Influential Factors for Scours Associated with *Cryptosporidium* sp., Rotavirus and Coronavirus in Calves from Argentinean Dairy Farms

Emiliano Bertoni [1], Adrián A. Barragán [2], Marina Bok [3], Celina Vega [3], Marcela Martínez [4], José F. Gil [5], Rubén O. Cimino [5] and Viviana Parreño [6,*]

[1] Área de Investigación en Salud Animal, IIACS-CIAP, INTA EEA Salta, Cerrillos A4403, Argentina; bertoni.emiliano@inta.gob.ar
[2] Veterinary Extension, Field Investigation & Research, Department of Veterinary and Biomedical Sciences, The Pennsylvania State University, State College, PA 16801, USA; axb779@psu.edu
[3] Instituto de Virología e INCUINTA, CICV y A, INTA Buenos Aires , Castelar 1712, Argentina; bok.marina@inta.gob.ar (M.B.); vega.celina@inta.gob.ar (C.V.)
[4] Área de Producción Animal, INTA EEA Salta, Cerrillos A4403, Argentina; martinez.gabriela@inta.gob.ar
[5] Cátedra de Química Biológica, Facultad de Ciencias Naturales, Universidad Nacional de Salta, Salta A4400, Argentina.; jgil.unsa@gmail.com (J.F.G.); rubencimino@gmail.com (R.O.C.)
[6] Instituto Nacional de Tecnología Agropecuaria, CICVyA, INCUINTA, Nicolas Repetto y de los Reseros s/n, Buenos Aires 1686, Argentina
* Correspondence: parreno.viviana@inta.gob.ar; Tel.: +54-011-3754 8400 (int 3364)

Citation: Bertoni, E.; Barragán, A.A.; Bok, M.; Vega, C.; Martínez, M.; Gil, J.F.; Cimino, R.O.; Parreño, V. Assessment of Influential Factors for Scours Associated with *Cryptosporidium* sp., Rotavirus and Coronavirus in Calves from Argentinean Dairy Farms. *Animals* **2021**, *11*, 2652. https://doi.org/10.3390/ani11092652

Academic Editors: Arcangelo Gentile and John Mee

Received: 24 May 2021

Accepted: 23 July 2021

Published: 09 September 2021

Publisher's Note: MDPI stays neutral with regard to jurisdictional claims in published maps and institutional affiliations.

Copyright: © 2021 by the authors. Licensee MDPI, Basel, Switzerland. This article is an open access article distributed under the terms and conditions of the Creative Commons Attribution (CC BY) license (http://creativecommons.org/licenses/by/4.0/).

Table S1. Risk factor associated with neonatal calf diarrhea in dairy farms from the Valle de Lerma, Salta, Argentina.

Variable	Category	Neonatal calf diarrhea rate (%), *p*-Value		Odds ratio (95% CI), *p*-Value
Herd size	Small	34 (64/188) [A]	0.0005	Reference category
	Median	32 (77/237) [A]		0.9 (0.6 - 1.4), 0.006
	Large	54 (32/59) [B]		2.2 (1.2- 4.2), 0.006
Dam vaccination	Yes	36 (163/446)	0.22	-
	No	26 (10/38)		
Time in calving pen (h)	<6	44 (64/146)	-	1.6 (1.7-2.4), 0.01
	>6	32.4 (111/342)		
Navel Desinfection	Yes	37 (142/382)	0.3	-
	No	31 (33/106)		
Colostrum intake	Dam	35 (110/311)	0.7	-
	Bottle or esophageal probe	37 (65/177)		
Volume of colostrum intake	Direct from the dam	35 (67/191)	0.3	Reference category
	<4 L	33.6 (70/208)		-
	>4 L	42 (38/89)		-
Colostrum bank	Yes	40.5 (103/254)	-	0.6 (0.4 – 0.9), 0.02
	No	30.7 (72/234)		
Calf rearing system	Individual	35.1 (98/280)	0.6	-
	Collective	36.5 (77/208)		
Liquid fedding	Milk replacer	40 (117/290)	-	1.6 (1 – 2.4), 0.01
	Raw milk	29 (58/198)		
Caretakers	One person	35 (164/461)	0.5	-
	Two persons	42 (11/27)		
	Female	33 (50/150)	0.5	-
	Male	36 (125/338)		
	Full time	41 (87/210)	-	0.6 (0.4 -0.9), 0.02
	Part time	31 (88/278)		
Diarrhea calf age	<20 days	63.23 (106/226)	-	2.4 (1.6 – 3.6), 0.0001
	>20 days	36.7 (69/262)		
RVA infection	Positive	58.6 (27/46)	-	2.8 (1.4 – 5.5), 0.001
	Negative	32.8 (148/442)		
C. sp infection	Positive	64 (64/100)	-	4.4 (2.7 – 7.2), 0.001
	Negative	28 (111/388)		

[A,B] Different letters indicate significant differences.

Table S2. Risk factor associated with RVA infection in dairy farms from the Valle de Lerma, Salta, Argentina.

Variable	Category	Bovine RVA infections rates, *p*-Value		Odds ratio (95% confidence interval), *p*-Value
Herd size	Small	7.4 (14/189)	0.3	Reference category
	Median	11 (27/238)		
	Big	8 (5/61)		
Dam vaccination	Yes	9.7 (44/450)	0.3	
	No	5 (2/38)		
Time in calving pen (H)	<6	6.1 (9/146)		0.5 (0.2-1.1), 0.1
	>6	10.8 (37/342)		
Navel desinfection	Yes	8.6 (34/382)	0.4	-
	No	11.3 (12/106)		
Colostrum system	Traditional system	8.7 (27/311)	0.5	-
	Artificial system	10.3 (19/177)		
Volume of colostrum in liters (L)	Traditional system	7.3 (14/191)	0.23	Reference category
	<4 L	12 (25/208)		
	>4 L	7.8 (7/89)		
Colostrum bank	Yes	9.4 (24/254)	1	-
	No	9.4 (22/234)		
Calf rearing system	Individual	6.7 (19/280)	-	0.5 (0.2 – 0.9), 0.02
	Collective	13 (27/208)	-	
Liquid fedding	Milk replacer	9.6 (28/290)	0.8	
	Raw milk	9 (18/198)		
Caretakers	One person	9.7 (45/461)	0.4	
	Two persons	3.7 (1/27)		
	Female	16 (24/150)	-	2.7 (1.4 - 5.3), 0.001
	Male	6.5 (22/338)		
	Full time	12.38 (26/210)		0.5 (0.2 – 1), 0.06
	Part time	7.22 (20/278)		
RVA infection age	<20 days	25 (31/226)	-	4.64 (2.27 – 9.49), 0.003
	>20 days	5.5 (15/262)		

Table S3. Risk factor associated with Cryptosporidium sp. infection in dairy farms from the Valle de Lerma, Salta, Argentina.

Variable	Categories	Bovine Cryptosporidium sp. infections rates, *p*-Value		Odds ratio (95% confidence interval), *p*-Value
Herd size	Small	17.59 (19/108) [A]	0.001	Reference category
	Median	13.75 (26/189) [A]		0.83 (0.4- 1.4), 0.03
	Big	30.13 (44/146) [B]		2.48 (1.2- 4.87), 0.03
Dam vaccination	No	28.9 (11/38)		1.65 (0.71, 3.6), 0.1
	Si	19.77 (89/405)		
Time in calving pen (Hs)	<6	23.28 (34/146)	0.3	
	>6	18.51 (55/297)		
Navel desinfection	Yes	20.94 (75/358)	0.8	-
	No	16.47 (14/85)		
Colostrum system	Traditional system	10.04 (37/205)		0.7 (0.42-1.5), 0.1
	Artificial system	21.84 (52/238)		
Volume of colostrum	Traditional system	15.87 (20/126) [A]	0.3	-
	<4 L	19.19 (38/198) [A]		
	>4 L	20.06 (31/119) [A]		
Colostrum bank	Yes	27.75 (68/245)	-	2.9 (1.7 – 4.8), 0.0001
	No	10.06 (21/198)		
Calf rearing system	Collective	27.13 (54/199)	-	0.5 (0.33 – .85), 0.005
	Individual	14.34 (35/244)		
Liquid fed	Raw milk	18.35 (29/158)	0.5	-
	Milk replacer	21.05 (60/285)		
Caretakers	One person	20.67 (86/416)	0.3	-
	Two persons	11.11 (3/27)		
	Male	21.95 (65/296)	-	0.7 (0.4 – 1.1), 0.1
	Female	16.10 (24/149)		
	Full time	25.71 (54/210)	-	0.5 (0.3 – 0.8), 0.007
	Part time	14.89 (35/235)		
Calves age (days)	<20	46.15 (57/117)	-	4.4 (2.6 – 7.5), 0.0001
	>20	7.6 (21/276)		

[A,B] Different letters indicate significant differences.
