# Supplementary material for: Assessment of Influential Factors for Scours Associated with Cryptosporidium sp., Rotavirus and Coronavirus in Calves from Argentinean Dairy Farms
Source: Animals (Basel). 2021 Sep 9;11(9):2652. doi: 10.3390/ani11092652 (PMC8466251; doi:10.3390/ani11092652)
Supplement: Supplementary file 1 [file animals-11-02652-s001.zip › animals-1244470-supplementary-part 2-Table S4- Statistics outputs of R.pdf]

animals

MDPI

Supplementary Materials

Assessment of Influential Factors for Scours Associated with *Cryptosporidium* sp., Rotavirus and Coronavirus in Calves from Argentinean Dairy Farms

Emiliano Bertoni [1], Adrián A. Barragán [2], Marina Bok [3], Celina Vega [3], Marcela Martínez [4], José F. Gil [5], Rubén O. Cimino [5] and Viviana Parreño [6,*]

[1] Área de Investigación en Salud Animal, IIACS-CIAP, INTA EEA Salta, Cerrillos A4403, Argentina; bertoni.emiliano@inta.gob.ar
[2] Veterinary Extension, Field Investigation & Research, Department of Veterinary and Biomedical Sciences, The Pennsylvania State University, State College, PA 16801, USA; axb779@psu.edu
[3] Instituto de Virología e INCUINTA, CICV y A, INTA Buenos Aires , Castelar 1712, Argentina; bok.marina@inta.gob.ar (M.B.); vega.celina@inta.gob.ar (C.V.)
[4] Área de Producción Animal, INTA EEA Salta, Cerrillos A4403, Argentina; martinez.gabriela@inta.gob.ar
[5] Cátedra de Química Biológica, Facultad de Ciencias Naturales, Universidad Nacional de Salta, Salta A4400, Argentina.; jgil.unsa@gmail.com (J.F.G.); rubencimino@gmail.com (R.O.C.)
[6] Instituto Nacional de Tecnología Agropecuaria, CICVyA, INCUINTA, Nicolas Repetto y de los Reseros s/n, Buenos Aires 1686, Argentina
* Correspondence: parreno.viviana@inta.gob.ar; Tel.: +54-011-3754 8400 (int 3364)

Citation: Bertoni, E.; Barragán, A.A.; Bok, M.; Vega, C.; Martínez, M.; Gil, J.F.; Cimino, R.O.; Parreño, V. Assessment of Influential Factors for Scours Associated with *Cryptosporidium* sp., Rotavirus and Coronavirus in Calves from Argentinean Dairy Farms. *Animals* **2021**, *11*, 2652. https://doi.org/10.3390/ani11092652

Academic Editors: Arcangelo Gentile and John Mee

Received: 24 May 2021

Accepted: 23 July 2021

Published: 09 September 2021

Publisher's Note: MDPI stays neutral with regard to jurisdictional claims in published maps and institutional affiliations.

Copyright: © 2021 by the authors. Licensee MDPI, Basel, Switzerland. This article is an open access article distributed under the terms and conditions of the Creative Commons Attribution (CC BY) license (http://creativecommons.org/licenses/by/4.0/).

Table S4. Statistics outputs of R.

Logistic regression procedure

The risk factor analysis was conducted using the Epical package (http://cran.r-project.org) in R Studio (Version 1.2.1335, © 2009-2019 RStudio, Inc.).

A first univariate screening of variables was conducted in order to identify them as risk factors associated to diarrhea, rotavirus infection and cp infection. The Epicalc function *"cc"* was applied, producing odds ratio, its 95% confidence interval, performing the Chi-squared ($\chi 2$) tests and Fisher's exact tests. The *'cc'* function uses the exact method to calculate the odds ratio (Epical Manual, https://usermanual.wiki/Document/EpicalcBook.934243917/view). Due to the low incidence of BCoV in the study population (2 calves positive) this variable was excluded from the analysis.

List of variables analyzed:

Case: diarrhea/healthy > calf with diarrhea or healthy calf at sampling.

HS: Herd size

Vac: Dam vaccination with vaccine to prevent calf scours

Tpc:Time of newborn in the calving pen

Do: Navel disinfection

Cna: Colostrum administration: traditional (natural) vs artifitial

Vc: Volume of colostrum administered

Cb:Colostrum bank

Gua: Calf rearing system, grouped or individual

Ld: Type of liquid diet, raw milk or milk replacer

CN Caretakers number

OT: Occupation time

CG: Caretaker gender

RVA: rotavirus

Cp: Cryptosporidium sp.

Age: Calf age

Univariate screening variables for scours

```
> cc(case, HS)
```

Variable / case	HS: Herd size		
	small	median	large
Diarrhea	65	77	33
Healthy	124	161	28
Odds ratio	1	1.1	0.45
lower 95% CI		0.72	0.24
upper 95% CI		1.68	0.83
Chi-squared = 10.272 , 2 d.f., P value = 0.006			
Fisher's exact test (2-sided) P value = 0.007			

```
> cc(case, vac)
```

Variable / case	VAC: Dam vaccination		
	YES	NO	total
Diarrhea	165	10	175
Healthy	285	28	28
Total	450	38	488
OR = 1.62	95%CI =0.77;3.42		
Chi-squared = 1.63, 1 d.f., P value = 0.201			
Fisher's exact test (2-sided) P value = 0.222			

```
> cc(case, Tpc)
```

Variable / case	Tpc: time of newborn in calving pen		
	<6h	>6h	total
Diarrhea	64	111	175
Healthy	82	231	313
Total	146	342	488
OR = 1.62 95%CI = 1.09, 2.42			

Chi-squared = 5.76, 1 d.f., P value = 0.016
Fisher's exact test (2-sided) P value = 0.018

```
> cc(case, Do)
```

Variable / case	Do: navel disinfection		
	YES	NO	total
Diarrhea	142	33	175
Healthy	240	73	313
Total	382	106	488
OR = 1.31 95%CI = 0.83, 2.07			
Chi-squared = 1.32, 1 d.f., P value = 0.251			
Fisher's exact test (2-sided) P value = 0.303			

```
> cc(case, cna)
```

Variable / case	Cna: colostrum administration		
	Traditional (Natural)	Artifitial	total
Diarrhea	110	65	175
Healthy	201	112	313
Total	311	177	488
OR =0.94 95% CI = 0.64, 1.38			

Chi-squared = 0.09, 1 d.f., P value = 0.764

Fisher's exact test (2-sided) P value = 0.769

```
> cc(case, Vc)
```

Variable / case	Vc: volume of colostrum administered		
	unknow	<4L	>4L
Diarrhea	67	70	38
Healthy	124	138	51
Odds ratio	1	1.07	0.73
lower 95% CI		0.69	0.42
upper 95% CI		1.65	1.26

Chi-squared = 2.299 , 2 d.f., P value = 0.317

Fisher's exact test (2-sided) P value = 0.312

```
> cc(case, Cb)
```

Variable / case	Cb: colostrum bank		
	YES	NO	total
Diarrhea	103	72	175
Healthy	151	162	313
Total	254	234	488
OR = 1.53 95%CI = 1.06, 2.23			
Chi-squared = 5.07, 1 d.f., P value = 0.024			

Fisher's exact test (2-sided) P value = 0.030

> cc(case, gua)

Variable / case	gua: calf rearing system		
	Grouped	individual	total
Diarrhea	77	98	175
Healthy	131	182	313
Total	208	280	488
OR = 1.09 95% CI = 0.75, 1.59			
Chi-squared = 0.21, 1 d.f., P value = 0.646			
Fisher's exact test (2-sided) P value = 0.703			

> cc(case, Ld)

Variable / case	Ld: Type of liquid diet, raw milk or milk replacer		
	Raw milk	Milk replacer	total
Diarrhea	58	117	175
Healthy	140	173	313
Total	198	290	488
OR = 0.61 95% CI = 0.42,0.9			
Chi-squared = 6.25, 1 d.f., P value = 0.012			
Fisher's exact test (2-sided) P value = 0.013			

```
> cc(case, CN)
```

Variable / case	CN: Number of Caretakers		
	Raw milk	Milk replacer	total
Diarrhea	164	11	175
Healthy	297	16	313
Total	461	27	488
OR = 0.80 95% CI = 0.36, 1.77			
Chi-squared = 0.3, 1 d.f., P value = 0.586			
Fisher's exact test (2-sided) P value = 0.680			

```
> cc(case, OT)
```

Variable / case	OT: occupation time		
	Part-time	Full-time	Total
Diarrhea	87	88	175
Healthy	123	190	313
Total	210	278	488
OR = 1.53 95% CI = 1.05, 2.22			
Chi-squared = 4.97, 1 d.f., P value = 0.026			
Fisher's exact test (2-sided) P value = 0.029			

```
> cc(case, CG)
```

Variable / case	CG: caretaker gender		
	Male	Female	total
Diarrhea	125	50	175
Healthy	213	100	313
Total	338	150	488
OR = 1.17	95% CI = 0.78, 1.76		
Chi-squared = 0.6, 1 d.f., P value = 0.438			
Fisher's exact test (2-sided) P value = 0.475			

```
> cc(case, RVA)
```

Variable / case	RVA: rotavirus		
	positive	negative	total
Diarrhea	27	148	175
Healthy	19	294	313
Total	46	442	488
OR = 2.82	95% CI =.52, 5.24		
Chi-squared = 11.51, 1 d.f., P value = 0.001			
Fisher's exact test (2-sided) P value = 0.001			

```
> cc(case, Cp)
```

Variable / case	Cp: Cryptosporidium sp.		
	positive	negative	total
Diarrhea	64	111	175
Healthy	36	277	313
Total	100	388	488

OR = 4.44 95% CI = 2.79, 7.06

Chi-squared = 43.3, 1 d.f., P value = 0

Fisher's exact test (2-sided) P value = 0

```
> cc(case, Age)
```

Variable / case	Age: Calf's age		
	[0,20]	(20,90]	total
Diarrhea	106	69	175
Healthy	120	193	313
Total	226	262	488

OR = 2.47 95% CI = 1.69, 3.61

Chi-squared = 22.31, 1 d.f., P value = 0

Fisher's exact test (2-sided) P value = 0

The variables with a P< 0.2 [11,26] in the univariate analysis were selected as explanatory effects for performing a Multivariable Logistic Regression (MLR) model where calf within farm was included in the model as a random effect. Using the Epicalc command *"logistic.display( )"* a display showing the OR (95% CI), P(Wald's test) and P(LR-test) was obtained. For the interpretation of the results it was considered the P-value that the results from Wald's test which depend on the reference level of the explanatory variable with only two categories, while the P-value from LR-test was considered only in the analysis of the variables with tree or more categories.

Logistic regression for the dependent variable scours

Case: diarrhea/healthy > calf with diarrhea or healthy calf at sampling.

HS: Herd size

Vac: Dam vaccination with vaccine to prevent calf scours

Tpc:Time of newborn in the calving pen

Do: Navel disinfection

Cna: Colostrum administration: traditional (natural) vs artifitial

Vc: Volume of colostrum administered

Cb:Colostrum bank

Gua: Calf rearing system, grouped or individual

Ld: Type of liquid diet, raw milk or milk replacer

CN Caretakers number

OT: Occupation time

CG: Caretaker gender

RVA: rotavirus

Cp: Cryptosporidium sp.

Age: Calf age

FULL MODEL

glm0 <- glm(case ~ HS + tpc + bc + Ld + OT + RVA + Cp + AGE, family=binomial)

> logistic.display(glm0)

Logistic regression predicting case : Diarrhea vs Healthy

	crude OR(95%CI)	adj. OR(95%CI)	P(Wald's test)	P(LR-test)
HS: ref.=small			0.006	
Medium	0.91 (0.61,1.37)	0.76 (0.43,1.33)	0.334	
Large	2.25 (1.25,4.04)	2.31 (0.94,5.64)	0.067	
tpc: >6h vs <6h	0.62 (0.41,0.92)	0.47 (0.25,0.9)	0.022	0.02
bc: No vs yes	0.65 (0.45,0.95)	1.43 (0.79,2.6)	0.237	0.232
Ld: m. replacer vs r. milk	1.63 (1.11,2.4)	1.92 (1.18,3.12)	0.009	0.008
OT: part vs Full Time	0.65 (0.45,0.95)	1.1 (0.66,1.83)	0.725	0.725

RVA: Positive vs Negative	2.82 (1.52,5.24)	2.73 (1.38,5.41)	0.004	0.004
Cp: Positive vs Negative	4.44 (2.79,7.06)	3.26 (1.94,5.47)	< 0.001	< 0.001
AGE: (20,90] vs [0,20]	0.4 (0.28,0.59)	0.44 (0.28,0.68)	< 0.001	< 0.001

Log-likelihood = -277.0623

No. of observations = 488

AIC value = 574.1246

The model selection was conducted by Stepwise selection of independent variables, we started with a full model including all variables selected with the univariate screening (p < 0.2). The command *step* removed each independent variable and compared the degrees of freedom reduced, the new deviance and the new AIC. The results were increasingly sorted by AIC. R selected the best fit model with the lowest Akaike Information Criterion (AIC) score. The variables of interest were considered significant if $P < 0.05$, and $P < 0.10$ was considered a tendency.

```
> modelstep <- step(glm0, direction="both")
```

```
Start:  AIC=574.12
caso ~ vo + tpc + bc + ls + to + rva + cp + ed
```

	Df	Deviance	AIC
- to	1	554.25	572.25
- bc	1	555.55	573.55
<none>		554.12	574.12
- tpc	1	559.52	577.52
- ls	1	561.09	579.09
- vo	2	564.27	580.27
- rva	1	562.55	580.55
- ed	1	567.88	585.88
- cp	1	574.77	592.77

```
Step:  AIC=572.25
caso ~ vo + tpc + bc + ls + rva + cp + ed
```

	Df	Deviance	AIC
- bc	1	555.73	571.73
<none>		554.25	572.25
+ to	1	554.12	574.12
- tpc	1	559.85	575.85
- ls	1	561.39	577.39
- rva	1	562.60	578.60
- vo	2	564.62	578.62
- ed	1	567.88	583.88
- cp	1	574.85	590.85

```
Step:  AIC=571.73
caso ~ vo + tpc + ls + rva + cp + ed
```

	Df	Deviance	AIC
<none>		555.73	571.73
+ bc	1	554.25	572.25
+ to	1	555.55	573.55
- tpc	1	559.86	573.86
- ls	1	562.22	576.22
- vo	2	564.85	576.85
- rva	1	563.83	577.83
- ed	1	569.80	583.80
- cp	1	574.96	588.96

```
> summary(modelstep)

Call:
glm (formula = case ~ HS + tpc + Ld + RVA + Cp + AGE, family = binomial)

Deviance Residuals:
    Min       1Q   Median       3Q
Max
-1.6880  -0.8082  -0.6176   0.9541   2.1
252

Coefficients:
             Estimate Std. Error z value Pr(>|z|)
(Intercept)  -0.3764     0.4069  -0.925 0.354954
HSMedian     -0.3703     0.2736  -1.353 0.175995
HSLarge       0.5767     0.3627   1.590 0.111843
tpc>6hs      -0.5749     0.2843  -2.022 0.043160 *
Ldreplacer    0.5879     0.2336   2.517 0.011852 *
rvaPositive   0.9895     0.3501   2.826 0.004713 **
cpPositive    1.1114     0.2556   4.348 1.37e-05 ***
age(20,90]    -0.8264     0.2220  -3.723 0.000197 ***
Signif. codes:  0 '***' 0.001 '**' 0.01 '*' 0.05 '.' 0.1 ' ' 1

(Dispersion parameter for binomial family taken to be 1)

Null deviance: 636.95  on 487  degrees of freedom
Residual deviance: 555.73  on 480  degrees of freedom
AIC: 571.73
```

Number of Fisher Scoring iterations: 4

> logistic.display(modelstep)

Logistic regression predicting case : Healthy vs Diarrhea

	crude OR(95%CI)	adj. OR(95%CI)	P(Wald's test)	P(LR-test)
HS: ref.=small			0.01	
Medium	1.1 (0.73,1.64)	1.45 (0.85,2.48)	0.176	
Large	0.44 (0.25,0.8)	0.56 (0.28,1.14)	0.112	
tpc: >6 h vs <6 h	1.62 (1.09,2.42)	1.78 (1.02,3.1)	0.043	0.042
ld: m.replacer vs r.milk	0.61 (0.42,0.9)	0.56 (0.35,0.88)	0.012	0.011
RVA: Negative vs Positive	2.82 (1.52,5.24)	2.69 (1.35,5.34)	0.005	0.004
Cp: Negative vs Positive	4.44 (2.79,7.06)	3.04 (1.84,5.01)	< 0.001	< 0.001
AGE: (20,90] vs [0,20]	2.47 (1.69,3.61)	2.29 (1.48,3.53)	< 0.001	< 0.001

Log-likelihood = -277.863

No. of observations = 488

AIC value = 571.7259

Univariate screening for the dependent variable rotavirus infection

```
cc(rva, HS)
```

Variable / rva	HS: Herd size small	median	large
Positive	14	27	5
Negative	175	211	56
Odds ratio	1	0.63	0.9
lower 95% CI		0.29	0.29
upper 95% CI		1.28	3.32
Chi-squared = 2.036 , 2 d.f., P value = 0.361			
Fisher's exact test (2-sided) P value = 0.381			

```
> cc(rva, VAC)
```

Variable / rva	VAC: Dam vaccination		
	YES	NO	total
Positive	44	2	46
Negative	406	36	442
Total	450	38	488
OR = 1.95 95% CI =0.45,8.38			
Chi-squared = 0.84, 1 d.f., P value = 0.361			
Fisher's exact test (2-sided) P value = 0.562			

```
> cc(rva, Tpc)
```

Variable / rva	Tpc: time of newborn in calving pen		
	<6h	>6h	total
Positive	9	37	46
Negative	137	305	442
Total	146	342	488
OR = 0.54 95% CI = 0.25, 1.15			
Chi-squared = 2.6, 1 d.f., P value = 0.107			
Fisher's exact test (2-sided) P value = 0.128			

```
> cc(rva, Do)
```

Variable / rva	Do: navel disinfection		
	YES	NO	total
Positive	34	12	46
Negative	348	94	442
Total	382	106	488
OR = 0.77 95% CI = 0.38, 1.54			
Chi-squared = 0.57, 1 d.f., P value = 0.451			
Fisher's exact test (2-sided) P value = 0.454			

```
> cc(rva, Cna)
```

Variable / rva	Cna: colostrum administration		
	Traditional (Natural)	Artifitial	total
Positive	27	19	46
Negative	284	158	442
Total	311	177	488
OR = 0.79 95% CI = 0.43, 1.47			
Chi-squared = 0.56, 1 d.f., P value = 0.45			
Fisher's exact test (2-sided) P value = 0.52			

```
> cc(rva, Vc)
```

Variable / rva	Vc: volume of colostrum administered		
	unknow	<4L	>4L
Positive	14	25	7
Negative	177	183	82
Odds ratio	1	0.58	0.93
lower 95% CI		0.27	0.33
upper 95% CI		1.2	2.82
Chi-squared = 2.875 , 2 d.f.,		P value = 0.237	
Fisher's exact test (2-sided)		P value = 0.258	

> cc(rva, Cb)

Variable / rva	Cb: colostrum bank		
	YES	NO	total
Positive	24	22	46
Negative	230	212	442
Total	254	234	488
OR = 1.01 95% CI = 0.55, 1.85			
Chi-squared = 0, 1 d.f.,		P value = 0.986	
Fisher's exact test (2-sided)		P value = 1	

```
> cc(rva, gua)
```

Variable / rva	gua: calf rearing system		
	Grouped	individual	total
Positive	27	19	46
Negative	181	261	442
Total	208	280	488
OR = 2.05 95% CI = 1.11, 3.8			
Chi-squared = 5.36, 1 d.f., P value = 0.021			
Fisher's exact test (2-sided) P value = 0.028			

```
> cc(rva, Ld)
```

Variable / rva	Ld: Type of liquid diet, raw milk or milk replacer		
	Raw milk	Milk replacer	total
Positive	18	28	46
Negative	180	262	442
Total	198	290	488
OR = 0.94 95% CI = 0.5, 1.74			
Chi-squared = 0.04, 1 d.f., P value = 0.834			
Fisher's exact test (2-sided) P value = 0.876			

```
> cc(rva, CN)
```

Variable / rva	CN: Number of Caretakers		
	One	Two	total
Positive	45	1	46
Negative	416	26	442
Total	461	27	488
OR = 2.81 95% CI = 0.37, 21.22			
Chi-squared = 1.1, 1 d.f.,	P value = 0.295		
Fisher's exact test (2-sided)	P value = 0.498		

```
> cc(case, OT)
```

Variable / rva	OT: occupation time		
	Part-time	Full-time	total
Positive	26	20	46
Negative	184	258	442
Total	210	278	488
OR = 1.82 95% CI = 0.99, 3.36			
Chi-squared = 3.77, 1 d.f.,	P value = 0.052		
Fisher's exact test (2-sided)	P value = 0.061		

```
> cc(case, CG)
```

Variable / rva	CG: caretaker gender		
	Male	Female	total
Positive	22	24	46
Negative	316	126	442
Total	338	150	488
OR = 0.37 95% CI = 0.2, 0.68			
Chi-squared = 10.96, 1 d.f.,	P value = 0.001		
Fisher's exact test (2-sided)	P value = 0.002		

```
> cc(case, Age)
```

Variable / rva	Age: Calf's age		
	[0,20]	(20,90]	total
Positive	31	15	46
Negative	195	247	442
Total	226	262	488
OR = 2.62 95% CI = 1.37, 4.99			
Chi-squared = 9.08, 1 d.f.,	P value = 0.003		
Fisher's exact test (2-sided)	P value = 0.003		

Logistic regression for the dependent variable rotavirus infection

```
glm1 <- glm(rva ~ gua + ot + gen + ed, family=binomial)
> logistic.display(glm1)
```

Logistic regression predicting rva : Positive vs Negative

	crude OR(95%CI)	adj. OR(95%CI)	P(Wald's test)	P(LR-test)
gua: Individual vs Group	0.49 (0.26,0.9)	0.47 (0.23,0.94)	0.033	0.032
ot: Part-time vs Full Time	0.55 (0.3,1.01)	1.12 (0.5,2.51)	0.784	0.784
Gen: Female vs Male	2.74 (1.48,5.06)	2.65 (1.23,5.7)	0.013	0.012
ed: (20,90] vs [0,20]	0.38 (0.2,0.73)	0.45 (0.23,0.88)	0.019	0.017

Log-likelihood = -141.469

No. of observations = 488

AIC value = 292.938

```
> modelstep <- step(glm1, direction="both")
Start:  AIC=292.94

rva ~ gua + to + sex + to + ed
```

	Df	Deviance	AIC
- ot	1	283.01	291.01
<none>		282.94	292.94
- gua	1	287.56	295.56
- ed	1	288.66	296.66
- gen	1	289.32	297.32

```
Step:  AIC=291.01

rva ~ gua + gen + ed
```

	Df	Deviance	AIC
<none>		283.01	291.01
+ ot	1	282.94	292.94
- gua	1	288.04	294.04
- ed	1	288.94	294.94
- gen	1	290.99	296.99

```
> summary(modelstep)

Call:

glm(formula = rva ~ gua + gen + ed, family = binomial)


Deviance Residuals:
```

Min	1Q	Median	3Q	Max

-0.7901	-0.5238	-0.3729	-0.2525	2.6312

Coefficients

	Estimate	Std. Error	z value	Pr(>|z|)
(Intercept)	-1.9172	0.2911	-6.587	4.5e-11 ***
guaIndividual	-0.7137	0.3213	-2.221	0.02633 *
genFmale	0.9129	0.3213	2.841	0.00449 **
ed(20,90]	-0.7988	0.3362	-2.376	0.01751 *

Signif. codes: 0 ‘***’ 0.001 ‘**’ 0.01 ‘*’ 0.05 ‘.’ 0.1 ‘ ’ 1

(Dispersion parameter for binomial family taken to be 1)

    Null deviance: 304.79 on 487 degrees of freedom

Residual deviance: 283.01 on 484 degrees of freedom

AIC: 291.01

Number of Fisher Scoring iterations: 5

> logistic.display(modelstep)

Logistic regression predicting rva : Positivo vs Negativo

	crude OR(95%CI)	adj. OR(95%CI)	P(Wald's test)	P(LR-test)
gua: Individual vs Group	0.49 (0.26,0.9)	0.49 (0.26,0.92)	0.026	0.025
Gen: Female vs Male	2.74 (1.48,5.06)	2.49 (1.33,4.68)	0.004	0.005

ed: (20,90] vs [0,20]	0.38 (0.2,0.73)	0.45 (0.23,0.87)	0.018	0.015

Log-likelihood = -141.5066
No. of observations = 488
AIC value = 291.0133

Univariate screening for the dependent variable cryptosporidium sp. infection

cc(rva, HS)

Variable / cp	HS: Herd size small	median	large
Positive	37	40	23
Negative	152	198	38
OR	1	1.2	0.4
lower 95% CI		0.71	0.21
upper 95% CI		2.04	0.8
Chi-squared = 13.174 , 2 d.f., P value = 0.001			
Fisher's exact test (2-sided) P value = 0.003			

```
> cc(cp, Vc)
```

Variable / Cp	Vc: volume of colostrum administered		
	unknow	<4L	>4L
Positive	45	39	16
Negative	146	169	73
Odds ratio	1	1.33	1.4
lower 95% CI		0.8	0.72
upper 95% CI		2.23	2.85
Chi-squared = 1.836 , 2 d.f.,		P value = 0.399	
Fisher's exact test (2-sided)		P value = 0.41	

```
> cc(cp, Tpc)
```

Variable / cp	Tpc: time of newborn in calving pen		
	<6h	>6h	total
Positive	34	66	100
Negative	112	276	388
Total	146	342	488
OR = 1.27 95% CI = 0.79, 2.03			
Chi-squared = 1, 1 d.f.,	P value = 0.317		
Fisher's exact test (2-sided)	P value = 0.329		

> cc(cp, Do)

Variable / cp	Do: navel disinfection		
	YES	NO	total
Positive	79	21	100
Negative	303	85	388
Total	382	106	488
OR = 1.06 95% CI = 0.62, 1.81			
Chi-squared = 0.04, 1 d.f., P value = 0.84			
Fisher's exact test (2-sided) P value = 0.89			

> cc(rva, Cna)

Variable / cp	Cna: colostrum administration		
	Traditional (Natural)	Artifitial	total
Positive	70	30	100
Negative	241	147	388
Total	311	177	488
OR = 1.42 95% CI = 0.89, 2.29			
Chi-squared = 2.14, 1 d.f., P value = 0.144			
Fisher's exact test (2-sided) P value = 0.162			

```
> cc(cp, Cb)
```

Variable / cp	Cb: colostrum bank		
	YES	NO	total
Positive	72	28	100
Negativq	182	206	388
Total	254	234	488
OR = 2.91 95% CI = 1.8, 4.7			
Chi-squared = 20.06, 1 d.f.,	P value = 0		
Fisher's exact test (2-sided)	P value = 0		

```
> cc(cp, gua)
```

Variable / cp	gua: calf rearing system		
	Grouped	individual	total
Positive	55	45	100
Negative	153	235	388
Total	208	280	488
OR = 1.88 95% CI = 1.2, 2.92			
Chi-squared = 7.88, 1 d.f.,	P value = 0.005		
Fisher's exact test (2-sided)	P value = 0.006		

```
> cc(cp, Ld)
```

Variable / cp	Ld: Type of liquid diet, raw milk or milk replacer		
	Raw milk	Milk replacer	total
Positive	38	62	100
Negative	160	228	388
Total	198	290	488
OR = 0.87 95% CI = 0.56, 1.37			
Chi-squared = 0.35, 1 d.f., P value = 0.55			
Fisher's exact test (2-sided) P value = 0.57			

```
> cc(rva, CN)
```

Variable / cp	CN: Number of Caretakers		
	One	Two	total
Positive	97	3	100
Negative	364	24	388
Total	461	27	488
OR = 2.13 95% CI = 0.63, 7.23			
Chi-squared = 1.54, 1 d.f., P value = 0.214			
Fisher's exact test (2-sided) P value = 0.325			

```
> cc(cp, OT)
```

Variable / cp	OT: occupation time		
	Part-time	Full-time	total
Positive	55	45	100
Negative	155	233	388
Total	210	278	488
OR = 1.84 95% CI = 1.18, 2.86			
Chi-squared = 7.35, 1 d.f.,	P value = 0.007		
Fisher's exact test (2-sided)	P value = 0.009		

```
> cc(cp, CG)
```

Variable / cp	CG: caretaker gender		
	Male	Female	total
Positive	75	25	100
Negative	263	125	388
Total	338	150	488
OR = 1.43 95% CI = 0.86, 2.35			
Chi-squared = 1.94, 1 d.f.,	P value = 0.163		
Fisher's exact test (2-sided)	P value = 0.182		

```
> cc(cp, Age)
```

Variable / cp	Age: Calf's age		
	[0,20]	(20,90]	total
Positive	74	26	100
Negative	152	236	388
Total	226	262	488
OR = 4.42 95% CI = 2.7, 7.22			
Chi-squared = 38.78, 1 d.f.,	P value = 0.0001		
Fisher's exact test (2-sided)	P value = 0.0001		

Logistic regression for the dependent variable Cryptosporidium sp. infection

```
> glm2 <- glm(cp ~ HZ + tpc + cb + ld + ot + ed, family=binomial)

> summary(glm2)


Call:

glm(formula = cp ~ HZ + tpc + cb + ld + ot + ed, family = binomial)
```

Deviance Residuals:

Min	1Q	Median	3Q	Max
-2.4249	0.2293	0.4242	0.6786	1.4035

Coefficients:

	Estimate	Std. Error	z value	Pr(>|z|)
(Intercept)	-0.1741	0.6795	-0.256	0.798
HZMedian	0.7397	0.4009	1.845	0.065 .
HZLarge	-0.1204	0.5656	-0.213	0.831
tpc>6 h	-0.1713	0.3661	-0.468	0.640
cbNo	1.4341	0.3409	4.207	2.59e-05 ***
Ldreplacer	-0.2228	0.3183	-0.700	0.484
Otpart-time	0.2626	0.3461	0.759	0.448
ed(20,90]	1.7574	0.2753	6.384	1.73e-10 ***

Signif. codes: 0 ‘***’ 0.001 ‘**’ 0.01 ‘*’ 0.05 ‘.’ 0.1 ‘ ’ 1

(Dispersion parameter for binomial family taken to be 1)

Null deviance: 494.97 on 487 degrees of freedom

Residual deviance: 414.55 on 480 degrees of freedom

AIC: 430.55

Number of Fisher Scoring iterations: 5

> logistic.display(glm2)

Logistic regression predicting cp : Negative vs Positive

	crude OR(95%CI)	adj. OR(95%CI)	P(Wald's test)	P(LR-test)
HZ: ref.=small				0.02
Median	1.2 (0.73,1.98)	2.1 (0.96,4.6)	0.065	
Large	0.4 (0.21,0.76)	0.89 (0.29,2.69)	0.831	
tpc: >6 h vs <6 h	1.27 (0.79,2.03)	0.84 (0.41,1.73)	0.64	0.641
cb: No vs Si	2.91 (1.8,4.7)	4.2 (2.15,8.18)	< 0.001	< 0.00
1				
ld: replacer vs milk	0.87 (0.56,1.37)	0.8 (0.43,1.49)	0.484	0.484
ot: Part-time vs Full-time	1.84 (1.18,2.86)	1.3 (0.66,2.56)	0.448	0.447
ed: (20,90] vs [0,20]	4.42 (2.7,7.22)	5.8 (3.38,9.94)	< 0.001	< 0.00
1				

Log-likelihood = -207.2758

No. of observations = 488

AIC value = 430.5515

```
> modelstep <- step(glm2, direction="both")
Start:  AIC=430.55
cp ~ HZ + tpc + cb + ld + ot + ed
```

	Df	Deviance	AIC
- tpc	1	414.77	428.77
- ld	1	415.04	429.04
- ot	1	415.13	429.13
<none>		414.55	430.55
- HZ	2	422.41	434.41
- cb	1	432.54	446.54
- ed	1	462.09	476.09

```
Step:  AIC=428.77
cp ~ HZ + cb + ld + ot + ed
```

	Df	Deviance	AIC
- ld	1	415.10	427.10
- ot	1	415.52	427.52
<none>		414.77	428.77
+ tpc	1	414.55	430.55
- HZ	2	425.83	435.83
- cb	1	433.56	445.56
- ed	1	462.52	474.52

```
Step:  AIC=427.1
cp ~ HZ + cb + ot + ed
```

	Df	Deviance	AIC
- ot	1	416.64	426.64
<none>		415.10	427.10
+ ld	1	414.77	428.77
+ tpc	1	415.04	429.04
- HZ	2	425.98	433.98
- cb	1	437.57	447.57
- ed	1	463.01	473.01

Step: AIC=426.64

cp ~ HZ + cb + ed

	Df	Deviance	AIC
<none>		416.64	426.64
+ ot	1	415.10	427.10
+ ld	1	415.52	427.52
+ tpc	1	416.54	428.54
- HZ	2	427.29	433.29
- cb	1	439.79	447.79
- ed	1	465.79	473.79

```
> summary(modelstep)

Call:
glm(formula = cp ~ HZ + cb + ed, family = binomial)

Deviance Residuals:
```

```
    Min       1Q   Median       3Q      Max
-2.4415   0.2256   0.4560   0.7052   1.3891
```

Coefficients:

	Estimate	Std. Error	z value	Pr(>|z|)	
(Intercept)	-0.2691	0.3067	-0.877	0.3803	
HZMedian	0.7302	0.3068	2.380	0.0173	*
HZlarge	-0.2161	0.4052	-0.533	0.5938	
cbNo	1.4473	0.3148	4.598	4.27e-06	***
ed(20,90]	1.7500	0.2698	6.487	8.73e-11	***

```
Signif. codes:  0 ‘***’ 0.001 ‘**’ 0.01 ‘*’ 0.05 ‘.’ 0.1 ‘ ’ 1

(Dispersion parameter for binomial family taken to be 1)

    Null deviance: 494.97  on 487  degrees of freedom
Residual deviance: 416.64  on 483  degrees of freedom
AIC: 426.64

Number of Fisher Scoring iterations: 5
```

```
> logistic.display(modelstep)

Logistic regression predicting cp : Negative vs Positive
```

	crude OR(95%CI)	adj. OR(95%CI)	P(Wald's test)	P(LR-test)
vo: ref.=Small				0.005
Median	1.2 (0.73,1.98)	2.08 (1.14,3.79)	0.017	
Large	0.4 (0.21,0.76)	0.81 (0.36,1.78)	0.594	
cb: No vs Yes	2.91 (1.8,4.7)	4.25 (2.29,7.88)	< 0.001	< 0.001
ed: (20,90] vs [0,20]	4.42 (2.7,7.22)	5.75 (3.39,9.76)	< 0.001	< 0.001

```
Log-likelihood = -208.3219
No. of observations = 488
AIC value = 426.6439
```
